# Lasso Peptides: Heterologous Production and Potential Medical Application

**DOI:** 10.3389/fbioe.2020.571165

**Published:** 2020-09-28

**Authors:** Cheng Cheng, Zi-Chun Hua

**Affiliations:** ^1^The State Key Laboratory of Pharmaceutical Biotechnology, School of Life Sciences, Nanjing University, Nanjing, China; ^2^School of Biopharmacy, China Pharmaceutical University, Nanjing, China; ^3^Changzhou High-Tech Research Institute of Nanjing University, Changzhou, China; ^4^Jiangsu Target Pharma Laboratories Inc., Changzhou, China

**Keywords:** lasso peptides, biosynthesis, heterologous expression, bioactivity, medical application

## Abstract

Lasso peptides are natural products found in bacteria. They belong to a specific family of ribosomally-synthesized and posttranslationally-modified peptides with an unusual lasso structure. Lasso peptides possess remarkable thermal and proteolytic stability and various biological activities, such as antimicrobial activity, enzyme inhibition, receptor blocking, anticancer properties and HIV antagonism. They have promising potential therapeutic effects on gastrointestinal diseases, tuberculosis, Alzheimer’s disease, cardiovascular disease, fungal infections and cancer. Lasso peptides with high stability have been shown to be good carriers for other bioactive peptides. These make them attractive candidates for pharmaceutical research. This review aimed to describe the strategies used for the heterologous production of lasso peptides. Also, it indicated their therapeutical potential and their capacity to use as an efficient scaffold for epitope grafting.

## Introduction

Lasso peptides are a class of ribosomally synthesized and posttranslationally modified peptides (RiPPs) (Maksimov et al., 2012a; Arnison et al., 2013). The C-terminal tail of a lasso peptide is trapped in the macrocyclic ring formed by seven to nine N-terminal amino acid residues. This interlocked topology confers them high thermal and proteolytic stability and distinguishes them from the other RiPPs.

Lasso peptide anantin was firstly identified in 1991 (Wyss et al., 1991) and microcin J25 (MccJ25) in 1992 in newborn feces (Salomon and Farias, 1992). Lariatin, with antimicrobial properties against mycobacteria including *Mycobacterium tuberculosis*, was discovered by activity-driven methods (Iwatsuki et al., 2007). Subsequently, in 1999, the gene cluster of the most well-studied MccJ25 was published (Solbiati et al., 1999), laying the foundation for studying the biosynthesis of lasso peptides. Marahiel and coll. identified the antimicrobial lasso peptide capistruin in 2008, which was first isolated using genome mining approaches (Knappe et al., 2008). Along with the development of the prokaryotic genome sequencing, the explosion in genome sequence data led to the development of genome-mining approaches to discover new lasso peptides (Maksimov et al., 2012b; Hegemann et al., 2013b; Tietz et al., 2017). Lasso peptides, especially specialicin (Kaweewan et al., 2018), achromosin (Kaweewan et al., 2017), sphaericin (Kodani et al., 2017), and actinokineosin (Takasaka et al., 2017), isolated from *Actinobacteria* exhibited antimicrobial activity.

Pandonodin that was identified from *Pandoraea norimbergensis* has the longest (18 residues) proteolytically resistant tail (Cheung-Lee et al., 2019a). Lasso peptides, burhizin-23, mycetohabin-16, and mycetohabin-15 were firstly isolated from endosymbiotic bacteria (Bratovanov et al., 2020). Huascopeptin, the most short-size lasso peptide, and leepeptin isolated from a cryptic *Streptomyces* gene cluster which represents a new family of lasso peptides, were both isolated from the extreme environment in the Salar de Huas, Atacama Desert, Chile (Gomez-Escribano et al., 2019; Cortes-Albayay et al., 2020).

Currently, it is expected that increasing numbers of lasso peptides will be discovered and characterized by genome-mining approaches (Tietz et al., 2017).

Lasso topology confers high stability against heat treatment, protease degradation, and extreme-pH environment. Hegemann (2019) described the examples of thermal sensitivity and stability of class II lasso peptides that were maintained by steric interaction. The influencing factors for the thermal stability of a lasso peptide include the size of the macrolactam ring, and the size and characteristics of the plug residue are critical in the heat stability of lasso peptides effectuated by mutational analysis (Knappe et al., 2009; Zimmermann et al., 2013, 2014; Hegemann et al., 2014, 2016). In general, the thermal stability increases with the increasing size of the plug residues and the decreasing size of the macrolactam ring (Hegemann, 2019). This review aimed to summarize the current state of thermal, enzymatic, and pH stability of the common lasso peptides, as shown in Table 1 (Knappe et al., 2009; Hegemann et al., 2013b, 2014; Zimmermann et al., 2013; Allen et al., 2016; Metelev et al., 2017; Tietz et al., 2017; Zong et al., 2017; Kodani et al., 2018; Martin-Gomez et al., 2018; Cheung-Lee et al., 2019b). Typically, the lasso topology endows the lasso peptides with excellent stability against thermally-induced unthreading and proteolysis (Maksimov and Link, 2013; Chekan et al., 2016; Fage et al., 2016; Hegemann, 2019), although not all peptides may have these properties. The combination of protease and heat treatment has been widely used to test the thermal stability of a lasso peptide. The use of carboxypeptidase Y, a protease that removes amino acids from the C-terminus of a peptide, is especially useful to detect when heat treatment induces the unthreading of a heat sensitive lasso peptide into a branched-cyclic peptide. Only a few studies investigated the pH stability of lasso peptides, MccJ25 was found to be stable against extreme pH (Yu et al., 2019). For example, capistruin (Knappe et al., 2008, 2009), MccJ25 (Yu et al., 2019), benenodin-1 (Zong et al., 2017), brevunsin (Kodani et al., 2018), and astexin-3 (Allen et al., 2016) exhibited remarkable stability against heat treatment. Among these, benenodin-1 established an equilibrium between two unique conformers upon heat treatment (Zong et al., 2017), proving it as a promising candidate for novel therapeutics because it could convert into an active conformation after administration and trigger a delayed response to the body temperature (Hegemann, 2019). MccJ25 can withstand high temperatures, such as 121°C, several proteases (pepsin, trypsin and chymotrypsin), and extremely low pH (2.0) (Naimi et al., 2018; Yu et al., 2020). However, MccJ25 appeared to have poor stability against elastase I one of the pancreatin components (Naimi et al., 2018).

**TABLE 1:** Thermal stability, enzymatic stability of commonly known lasso peptides.

Lasso peptides	Heat validation		Enzymatic validation		References
Astexin-1	No	20, 35, 50, 65, and 80°C, 95°C* for 1, 2, 4, and 8 h	No	Carboxypeptidase Y	Zimmermann et al., 2013
Astexin-2	No		No	Carboxypeptidase Y	Allen et al., 2016
Astexin-3	Yes		No	Carboxypeptidase Y	Allen et al., 2016
Acinetodin	Yes	95°C* for 4 h	Yes	Carboxypeptidase Y	Metelev et al., 2017
Benenodin-1	Yes	35–95°C* for 18 h	Yes	Carboxypeptidase B and Y	Zong et al., 2017
Brevunsin	Yes	50, 65, 80, and 95°C* for 1 h	–	–	Kodani et al., 2018
Burhizin	Yes	95°C* for 1 h	Yes	Carboxypeptidase Y	Hegemann et al., 2013b
Capistruin	Yes	95°C* for 1 h	–	–	Knappe et al., 2009
Caulosegnin I Caulosegnin II Caulosegnin III	No Yes No	20, 35, 50, 65, and 80°C for 4 h or 95°C* for 1, 2, 4, or 8 h	Yes Yes Yes	Trypsin Chymotrypsin Elastase Proteinase K Carboxypeptidase Y	Hegemann et al., 2013a
Chaxapeptin	Yes	95°C* for 1, 2, 3, or 4 h	Yes	Trypsin Chymotrypsin Epsin Papain Thermolysin Carboxypeptidase Y	Martin-Gomez et al., 2018
Citrocin	Yes	95°C* for 3 h	Yes	Carboxypeptidase Y	Cheung-Lee et al., 2019b
Citrulassin A	Yes	95°C* for 4 h	Yes	Carboxypeptidase Y	Tietz et al., 2017
Caulonodin I Caulonodin II Caulonodin III	Yes Yes Yes	95°C* for 1 h	Yes No Yes	Carboxypeptidase Y Carboxypeptidase Y Carboxypeptidase Y	Hegemann et al., 2013b
Klebsidin	Yes	95°C* for 4 h	Yes	Carboxypeptidase Y	Metelev et al., 2017
Microcin J25	Yes	25, 37, 45, 55, 65, 75, 85, and 95°C for 20 min or 121°C* for 20 min	Yes	Pepsin Trypsin Chymotrypsin	Yu et al., 2019
Rhodanodin	No	95°C for 1 h	No	Carboxypeptidase Y	Hegemann et al., 2013b
Rubrivinodin	No	95°C* for 1 h	Yes	Carboxypeptidase Y	Hegemann et al., 2013b
Sphingonodin I Sphingonodin II	Yes No	95°C* for 1 h	No No	Carboxypeptidase Y	Hegemann et al., 2013b
Syanodin I	No	95°C* for 1 h	No	Carboxypeptidase Y	Hegemann et al., 2013b
Sphingopyxin I Sphingopyxin II	No Yes	95°C* for 1 h	No No	Carboxypeptidase Y	Hegemann et al., 2013b
Xanthomonin I Xanthomonin II Xanthomonin III	Yes Yes Yes	95°C * for 8 h	No Yes Yes	Carboxypeptidase Y Chymotrypsin Proteinase K	Hegemann et al., 2014

*The highest temperature is marked with^∗^ in the heat validation.*

Lasso peptides have gained increasing attention owing to their stability under harsh conditions and amenability to functional engineering (Martin-Gomez and Tulla-Puche, 2018). This review comprehensively summarizes the current knowledge about the strategies of heterologous expression and potential medical application of lasso peptides.

## Structural Classifications and Biosynthesis of Lasso Peptides

**FIGURE 1:**
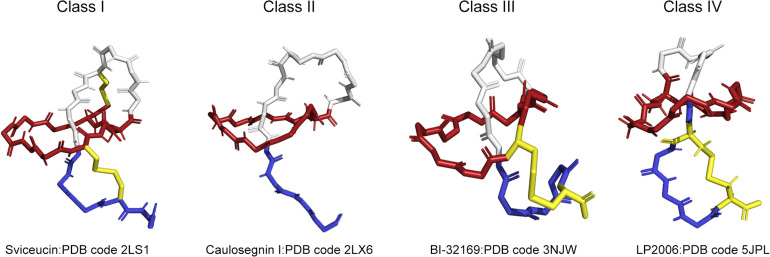
Four types of lasso peptides demonstrated by representative structures (class I, class II, class III, and class IV). Sviceucin: PDB code 2LS1 for class I, caulosegnin I: PDB code 2LX6 for class II, BI-32169: PDB code 3NJW for class III, LP2006: PDB code 5JPL for class IV. The macrolactam ring is colored in red, the tail is in blue, the disulfide bonds are in yellow.

According to the presence, number and location of disulfide bridges, four classes of lasso peptides have been defined and are shown in Figure 1. A better understanding of the structure of lasso peptides may lay a significant foundation for the discovery of their roles and application as therapeutics. Lasso peptides containing two disulfide bonds are defined as class I. Lasso peptides that do not contain any disulfide bonds and whose topology in turn is only stabilized by steric interactions pertain to class II. Lasso peptides that have one disulfide bond belong to class III or class IV. The disulfide bond of class III lasso peptides forms between the macrocyclic ring and the tail, while that of class IV is formed in the tail. Specifically, the disulfide bridge formed in the tails of class IV results in a plug that prevents the tail being unthreaded. Most of the so far studied class II lasso peptides originated from *Proteobacteria*, while all lasso peptides belonging to classes I, III, and IV were thus far isolated from *Actinobacteria*. In addition, the classification of lasso peptides is related to their hydrophilicity. Except MccJ25, propeptin and anantin; most of the class II lasso peptides are hydrophilic (Maksimov and Link, 2014). The precursor peptide (A) of lasso peptides is composed of the N-terminal leader peptide region and the C-terminal core peptide region. The N-terminal leader peptide is responsible for the substrate recognition and the interaction between the post-translational processing enzymes, while the core peptide is where the posttranslational modifications are introduced. Among most of the isolated lasso peptides, Gly, Ser, Cys, or Ala are common at the N-terminus (Hegemann et al., 2013a; Metelev et al., 2015). Strikingly, the preferred residues at the first core position are small residues, such as Gly (62%) or Ala (16%), although about 7% lasso peptides are characterized by Leu at the N-terminus (Pan and Link, 2011; Tietz et al., 2017; Hegemann et al., 2018). A RiPP recognition element (RRE/E) expressed in the biosynthetic gene clusters of lasso peptides binds the leader peptide and directs the posttranslational modification. The conserved Thr residue at the penultimate position in the leader peptide acts as the recognition site for the maturation machinery of lasso peptides (Pan et al., 2012b; Hegemann et al., 2018).

**FIGURE 2:**
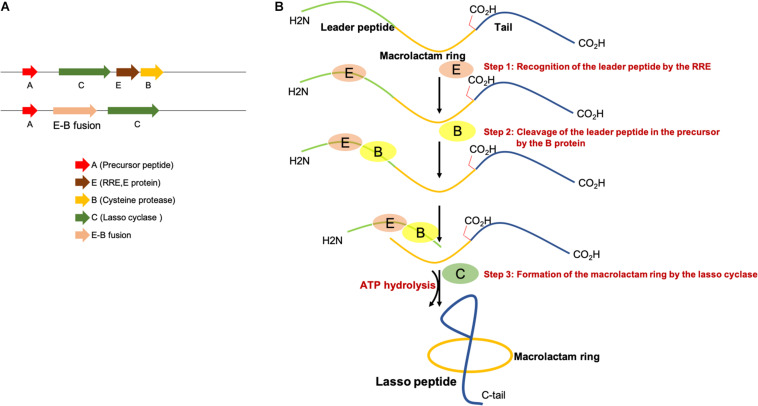
**(A)** Representative lasso peptide biosynthetic gene clusters with a separate E and B protein or E-B fusion. **(B)** Proposed mechanism of lasso peptide biosynthesis, involving three steps.

Representative lasso peptide biosynthetic gene clusters encoding separate RRE/E and the cysteine protease (B) or E-B fusions are shown in Figure 2A. Three steps are required for the biosynthesis of lasso peptides. First, the leader peptide in the precursor peptide (A) is recognized and bound by the RRE and then B protein removes the leader peptide by proteolysis and thereby releases the core peptide. Next, the lasso cyclase (C) activates the Asp/Glu carboxylic acid in the form of an AMP ester before catalyzing the macrolactam formation by condensation with the α-amino group as shown in Figure 2B. The ABC transporters (D) encoded in some lasso peptide biosynthetic gene clusters perform the export of lasso peptides out of the cells, and the presence of a D gene in a gene cluster indicates that the produced lasso peptides could potentially exhibit antibacterial activity (Choudhury et al., 2014; Hegemann et al., 2015; Bountra et al., 2017; Romano et al., 2018). Most of the *Proteobacterial* gene clusters without ABC-transporter carry an isopeptidase gene (*isoP*). Maksimov et al. (2015) firstly isolated an *isoP* from the gene clusters of astexin-2 and -3 (Zimmermann et al., 2013). The MS analysis revealed that IsoP produces linear peptides by hydrolyzing the isopeptide bond. However, the isopeptide bond of other lasso peptides, including branched-cyclic astexin-2 and -3 remained intact with IsoP treatment (Maksimov et al., 2015). This indicated that IsoPs specifically hydrolyze the isopeptide bond in the lasso peptides produced by adjacent clusters (Chekan et al., 2016; Fage et al., 2016). The structures of RREs from different RiPP families are comprised of three N-terminal β-strands and three C-terminal α-helices (Burkhart et al., 2015; Zhu et al., 2016a; Hegemann et al., 2018; Chekan et al., 2019; DiCaprio et al., 2019; Hegemann, 2019; Koos and Link, 2019; Sumida et al., 2019).

**FIGURE 3:**
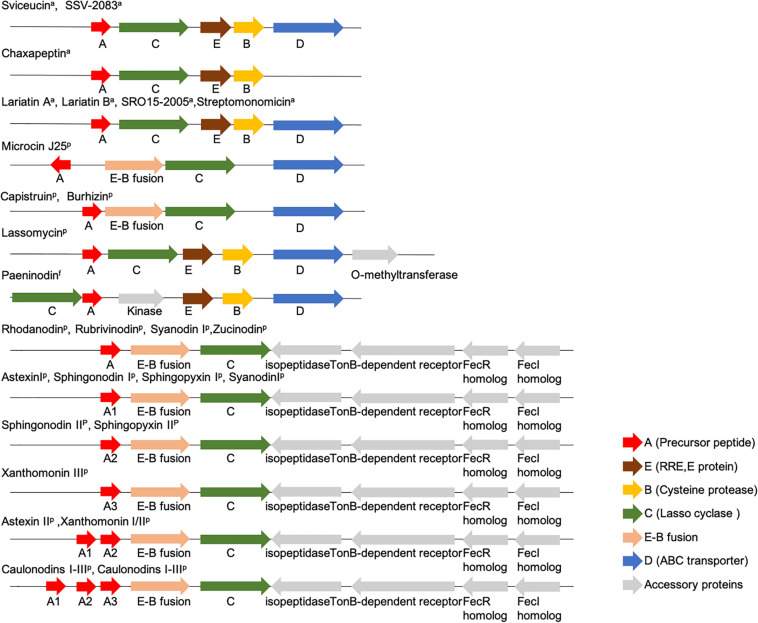
Organization of commonly known lasso peptides from *Actinobacteria*, *Proteobacteria*, and *Firmicutes* (Solbiati et al., 1999; Knappe et al., 2008; Inokoshi et al., 2012; Maksimov et al., 2012b; Hegemann et al., 2013b, 2014; Maksimov and Link, 2013; Zimmermann et al., 2014; Zhu et al., 2016a; Kodani et al., 2018; Martin-Gomez et al., 2018; Su et al., 2019; Yu et al., 2020).

Lasso peptides are derived from gene-encoded precursor peptides. The genetic organization of the commonly known lasso peptides from *Actinobacteria*, *Proteobacteria*, and *Firmicutes* is shown in Figure 3 (Solbiati et al., 1999; Knappe et al., 2008; Inokoshi et al., 2012; Maksimov et al., 2012b; Hegemann et al., 2013a, b, 2014; Maksimov and Link, 2013; Zimmermann et al., 2013, [Bibr B101][Bibr B62]; Zhao et al., 2016; Su et al., 2019). Notably, the E-B fusion occurs only in a subset of *Proteobacteria*, while discrete E and B proteins are found in *Firmicutes* and *Actinobacteria*.

## Heterologous Expression of Lasso Peptides

The biosynthesis of lasso peptides is designated by gene clusters, normally encoding A and C proteins and separated E and B proteins or E-B fusion (Hegemann, 2019). The homologous production of lasso peptides relies on the native growth conditions in the habitat. The close simulation of growth conditions is significant in the discovery of natural products (Knappe et al., 2008). Therefore, the homologous production of lasso peptides is hard to achieve under the standard culture conditions. The isolation of these natural products from native organism is often impractical (Maksimov et al., 2012b) because one strain may produce many kinds of natural products, such as *Streptomyces* species (Hertweck, 2009). Heterologous expression is a useful tool for the production of poorly expressed or silent lasso peptide biosynthetic gene clusters. For example, astexin-1 is not detected in *Asticcacaulis excentricus* under laboratory conditions, while heterologous expression was successfully achieved in *Escherichia coli* (Maksimov et al., 2012b). Additionally, heterologous expression might significantly improve the yield of lasso peptides under optimized cultivation conditions, facilitating the purification and structural studies. The heterologous production of lasso peptide brevunsin from *Brevundimonas diminuta* was achieved with a yield of 10.2 mg/L in *Sphingomonas subterranea* (Kodani et al., 2018). The genes involved in the biosynthesis and posttranslational modification have become accessible with the development of the functional study of lasso peptides (Pan et al., 2012a; Burkhart et al., 2015; Li et al., 2015; Zhu et al., 2016b; Hegemann et al., 2018; Chekan et al., 2019; DiCaprio et al., 2019; Koos and Link, 2019; Sumida et al., 2019). The heterologous expression of lasso peptides has gained increasing attention, and significant progress has been made in the production, especially based on the *E. coli* and *Streptomyces* systems. The common strategies of heterologous expression of lasso peptides are shown in Table 2.

**TABLE 2:** Heterologous expression of commonly known lasso peptides.

Lasso peptides	Native host	Heterologous host	Strategies	Yields	Location	References
Astexin-1	*A. excentricus*	*E. coli* BL21 Gold	RBS substitution/removal of the hairpin	–	Culture medium	Zimmermann et al., 2013
Astexin-2/-3	*A. excentricus*	*E. coli* BL21 Gold	RBS substitution	–	Cell lysate	Maksimov and Link, 2013
Albusnodin	*S. albus* DSM 41398	*S. coelicolor* M1146 *S. lividans* 66	Promoter engineering	–	Culture medium and lysate extracts	Zong et al., 2018
Benenodin-1/-2	*A. benevestitus*	*E. coli* BL21 Gold	RBS substitution/Promoter engineering	–	Cell lysate	Zong et al., 2017
Brevunsin	*B. diminuta*	*S. subterranea*	Promoter engineering	10.2 mg/L	Culture medium	Kodani et al., 2018
Capistruin	*B. thailandensis* E264	*E. coli* BL21 Gold	Optimization of culture conditions (defined medium M20)	0.2 mg/L	Culture medium Culture medium	Knappe et al., 2008
		*E. coli* strain XL-1 Blue	RBS substitution/secondary structure modification	1.6 mg/L		Pan et al., 2012b
		*Burkholderia* sp. FERM BP-3421	Optimization of culture conditions (2S4G medium)/Deletion of the autologous spliceostatin gene cluster	13 mg/L (One wild-type FERM BP-3421 outlier produced 116 mg/L)	Culture medium	Kunakom and Eustaquio, 2020
Caulosegnin I Caulosegnin II Caulosegnin III	*C. segnis*	*E. coli* BL21	RBS substitution/separation of gene encoding	∼0.30 mg/L ∼0.15 mg/L ∼0.10 mg/L	Culture medium	Hegemann et al., 2013a
Caulonodin I Caulonodin II Caulonodin III	*Caulobacter* sp. K31	*E. coli* BL21 Gold	RBS substitution	3.4 mg/L 2.2 mg/L 0.5 mg/L	Cell pellet extracts	Hegemann et al., 2013b
Caulonodins IV–VII	*Caulobacter* sp. K31	*E. coli* BL21 (DE3)	RBS substitution	0.7 mg/L ∼3-4 mg/L 10 mg/L	Cell pellet extracts	Zimmermann et al., 2014
Chaxapeptin	*S. leeuwenhoekii*	*E. coli* BL21 (DE3)	RBS substitution/optimization of culture conditions	∼0.1 mg/L	Culture medium	Martin-Gomez et al., 2018
Citrulassin A	*S. albulus*	*S. lividans* 1F3, *S. lividans* 3H4	–	–	Culture medium	Tietz et al., 2017
Citrocin	*C. pasteurii* and *C. braakii*	*E. coli* BL21	Codon optimization/promoter engineering	2.7 mg/L	Culture medium	Cheung-Lee et al., 2019b
Microcin J25/MccJ25 UAA	*E. coli* AY25	*E. coli* XL-1 Blue/*E. coli* BL21 Gold	Promoter engineering	4–16 mg/L/∼10–30% of wild-type MccJ25 production	Culture medium	Pan et al., 2010; Pan and Link, 2011; Piscotta et al., 2015; Zhang et al., 2018
Pandonodin	*P. norimbergensis*	*E. coli* BL21	Promoter engineering	2 mg/L	Culture medium	Cheung-Lee et al., 2019a
Rubrivinodin	*R. gelatinosus* IL44	*E. coli* BL21 Gold	RBS substitution	0.5 mg/L	Cell pellet extracts	Hegemann et al., 2013b
Sphingonodin I Syanodin I Sphingopyxin I Sphingopyxin II	*S. japonicum* UT26	*E. coli* BL21 Gold	RBS substitution	0.9 mg/L 5.2 mg/L 3.4 mg/L 0.4 mg/L	Cell pellet extracts	Hegemann et al., 2013b
Sviceucin	*S. sviceus* DSM 924T	*S. coelicolor* M1146	Site-specific integration	15 mg/L	Mycelia and culture supernatants	Li et al., 2015
Snou-LP 9401-LP1 9810-LP	*S. noursei* ATCC 11455 *Streptomyces* sp. ADI94-01 *Streptomyces* sp. ADI98-10	*S. albulus* J1074, *S. lividans* TK24	Construction of an orthogonal SARP-based expression system/optimization of culture conditions	0.8 mg/L Promising for scaling-up Promising for scaling-up	Cell pellet extracts	Mevaere et al., 2018

*A. excentricus, Asticcacaulis excentricus; E. coli, Escherichia coli; S. albus, Streptomyces albus; S. coelicolor, Streptomyces coelicolor; S. lividans, Streptomyces lividans; A. benevestitus, Asticcacaulis benevestitus; B. diminuta, Brevundimonas diminuta; S. subterranea, Sphingomonas subterranea; B. thailandensis, Burkholderia thailandensis; C. segnis, Caulobacter segnis; S. leeuwenhoekii, Streptomyces leeuwenhoekii; C. pasteurii, Citrobacter pasteurii; C. braakii, Citrobacter braakii; P. norimbergensis, Pandoraea norimbergensis; R. gelatinosus, Rubrivivax gelatinosus; S. japonicum, Sphingobium japonicum; S. sviceus, Streptomyces sviceus; S. noursei, Streptomyces noursei).*

### Heterologous Expression of Lasso Peptides in *E. coli*

The production of lasso peptides from *Proteobacteria* in *E. coli* is plausible. Due to appropriate regulatory elements and weak promoters, the heterologous production of lasso peptides from Gram-negative *Proteobacteria* in *E. coli* often has higher yields than homologous production through the fermentation of native producers. Firstly, selecting optimal culture conditions, such as temperature, components of the culture medium, and pH, is crucial for the production of lasso peptides. The highest yield of MccJ25 in *E. coli* was achieved in minimal, nutrient-poor media (Salomon and Farias, 1994), where the production of MccJ25 in *E. coli* begins after the bacteria enter the stationary phase (Salomon and Farias, 1992; Chiuchiolo et al., 2001). This phenomenon could be attributed to the fact that MccJ25-producing cells can thereby kill competitors in the same environment under nutrient-limiting conditions (Pan et al., 2010). Reportedly, the production of secondary metabolites (Salomon and Farias, 1994; Bibb, 2005), and microcins, was positively linked to the richness of the media. MccJ25 from the natural cluster is not produced in the rich media such as LB and only in minimal media (Salomon and Farias, 1992; Chiuchiolo et al., 2001). However, an engineered gene cluster was constructed for the production of MccJ25, which was neither dependent on the growth phase of the cell culture nor the media composition (Pan et al., 2010). The production of capistruin from *Burkholderia thailandensis* E264 increased by 300-fold in the M20 medium at 42°C as compared with that under initial screening conditions (M9 medium, 0.2% arabinose, 37°C, 24 h), achieving a yield of 0.2 mg/mL, while none was produced in the LB medium (Knappe et al., 2008). Secondly, introduction of one or more constitutive or inducible promoters was also shown to be effective. Two orthogonally inducible promoters were constructed in the expression system to permit a separate control of the production and the export/immunity of lasso peptide MccJ25 in *E. coli*, resulting in the high-throughput discovery of functional MccJ25 variants with multiple amino acid substitutions (Pan and Link, 2011). The relative production levels of these variants were approximately 0.5- to 2-fold of that of wild-type MccJ25 (8 mg/L) (Pan et al., 2010; Pan and Link, 2011). The gene cluster of capistruin was engineered under the control of an inducible tetracycline promoter, and the short sequence containing the *E. coli* Shine-Dalgarno sequence (AGGAGA) was used to replace the intergenic region between *capA* and *capB*. The yield of capistruin (1.6 mg/L) was increased by at least 8-fold compared with the previously reported heterologous expression system, and was two fold higher than the native producer strain yield (Pan et al., 2012b). The citrocin gene cluster, being refactored by placing *citA* under the IPTG-inducible T5 promoter while placing the *citBCD* genes under the control of a constitutive promoter, was heterologously expressed in *E. coli* with the yield of 2.7 mg/L (Cheung-Lee et al., 2019b). Two approaches were adopted for the heterologous expression of the gene cluster of pandonodin, yielding approximately 2 mg/L of culture. Firstly, the gene cluster *panABCD* was under an inducible *tet* promoter. Secondly, the *panA* gene was engineered under an inducible T5 promoter, while the intact putative operon *panBCD* was engineered under the control of a constitutive promoter (Cheung-Lee et al., 2019a). Thirdly, the incorporation of an *E. coli* optimized RBS also significantly increased the yields of other lasso peptides, like caulosegnins I–III (Hegemann et al., 2013a), astexin-1 (Zimmermann et al., 2013), astexin-2, astexin-3 (Maksimov and Link, 2013). For the optimization of heterologous lasso peptide production from clusters with more than one precursor peptide [e.g., the clusters of xanthomonins I-II (Hegemann et al., 2014), caulosegnins I-III (Hegemann et al., 2013a), caulonodins I-III (Hegemann et al., 2013a), caulonodins IV-V (Zimmermann et al., 2014), caulonodins VI-VII (Zimmermann et al., 2014), astexins-2 and -3 (Maksimov and Link, 2013)], it is important to not only replace the intergenic regions between the genes encoding precursor peptides and B protein with an artificial ribosomal binding site (RBS), but to also generate production plasmids encoding only a single precursor peptide. This approach not only increases yields of each individual lasso peptide, but also facilitates the purification of these compounds. The yield of caulonodin VII was 0.7 mg/L, that of caulonodins IV and V was 3-4 mg/L, and that of VI was 10 mg/L culture (Zimmermann et al., 2014). The native benenodin-1 gene cluster with a *tet* promoter and upstream RBS was expressed in *E. coli* (Zong et al., 2017), and the gene encoding the precursor was separated in the caulosegnin system to further improve the yields of lasso peptides (Hegemann et al., 2013a). The production of 12 lasso peptides was improved by the incorporation of RBS, especially 84.5-fold for caulonodin I (Hegemann et al., 2013b).

### Heterologous Expression of Lasso Peptides in *Streptomyces* and *Bacillus subtilis*

*E. coli* is not always applicable for the heterologous expression of lasso peptides, lassomycin-like lasso peptides derived from *Sanguibacter keddieii* DSM 10542 and *Streptomyces* sp. Amel2xC10 were poorly expressed in *E. coli* (Su et al., 2019). Gram-positive bacteria have also been widely used in the production of lasso peptides owing to their secretion ability, the high GC content of the promoters, and effective production of metabolic products. *Streptomyces* produce many useful products, including antibiotics and a large number of secreted enzymes (Pan et al., 2010), Recently, *Streptomyces* have shown their significant potential to produce novel natural products, Myronovskyi and Luzhetskyy (2019) have detailedly discussed the heterologous production of natural products in streptomycetes, including the genetic control elements developed for heterologous expression of biosynthetic gene clusters and the most widely used *Streptomyces* hosts. This work focuses on the successful heterologous expression of lasso peptides in streptomycetes. For example, an orthogonal system was constructed for the heterologous expression of lasso peptides in *Streptomyces albus* and *Streptomyces lividans*, based on the regulatory circuit from *Actinoalloteichus fjordicus.* Three lasso peptides including 9401-LP1, 9810-LP, and Snou-LP were excreted in the medium. A yield of 0.8 mg/L of Snou-LP was obtained after the scale-up production and purification in the solid SFM culture (Mevaere et al., 2018). Citrulassin A was expressed in *S. lividans* by constructing two fosmids covering about 20 kb upstream and downstream of its native biosynthetic gene cluster (Tietz et al., 2017). The heterologous expression of acetylated lasso peptides also required a *Streptomyces* host. Lasso peptide, albusnodin, encoded in the genome of *S albus* DSM 41398 is heterologously expressed in the hosts *Streptomyces coelicolor* and *S. lividans* (Zong et al., 2018). Sviceucin originating from *Streptomyces sviceus* is heterologously produced in good yield (15 mg/L of culture) in *S. coelicolor* (Li et al., 2015). The heterologous expression of biosynthetic gene clusters of Lp3 (chaxapeptin) and Lp2 (leepeptin) from *Streptomyces leeuwenhoekii* C34^*T*^ were achieved in *S. coelicolor* (Gomez-Escribano et al., 2019). For lassomycin, due to the lack of biosynthetic information and the abortive attempts on heterologous expression in *E. coli*, there are no analogs that have been reported (Gavrish et al., 2014; Hegemann et al., 2015; Su et al., 2019), the *Streptomyces*-based production system might be a preferable biosynthetic platform to generate methylated lasso peptides (Su et al., 2019). Currently, the most widely applied *Streptomyces* host for the production of lasso peptides are *S. coelicolor*, *S. lividans*, and *S. albus* (Tietz et al., 2017; Mevaere et al., 2018).

Another important group of Gram-positive heterologous hosts is *B. subtilis* (Zhang et al., 2016). *Burkholderia* genomes contain up to 27 biosynthetic gene clusters, encoding compounds from diverse biosynthetic classes (Kunakom and Eustaquio, 2019). Moreover, *Burkholderia* has higher G + C DNA content (∼67%) than *E. coli* (∼50%). Recently, higher yields of capistruin were obtained in the *Burkholderia* host than previously reported in *E. coli*. An unprecedented capistruin amount (up to 116 mg/L) was obtained with *Burkholderia* sp. FERM BP-3421, with a yield increased by 580-fold over *E. coli* (Kunakom and Eustaquio, 2020).

## Bioactivities of Lasso Peptides

### Antimicrobial Activity

As one kind of bacteriocins with unique lasso topology, some lasso peptides can kill other bacteria, which are closely or distantly related to the producing bacteria. The majority of antimicrobial lasso peptides exhibited antibacterial property against a wide range of pathogens (Table 3). Various lasso peptides act on Gram-negative bacteria. For instance, MccJ25 exhibits antibacterial activity against *Salmonella*, *Shigella flexneri*, *E. coli*, and *Enterobacter bugandensis* (Salomon and Farias, 1992; Pati et al., 2018). Anantin B1/B2 also shows weak antibacterial activity against *E. coli*. The lasso peptides, chaxapeptin, LP2006, anantin B2, sviceucin, streptomonomicin are active against Gram-positive bacteria. Chaxapeptin has comparatively weak antibacterial activity against *Staphylococcus aureus* and *B. subtilis* with the minimal inhibitory concentration (MIC) being 30–35 μg/mL (Elsayed et al., 2015). Capistruin showed weak antibacterial activity against *Burkholderia caledonica*, *Burkholderia caribensis*, *Burkholderia ubonensis*, *Burkholderia vietnamiensis*, *E. coli*, and *Pseudomonas aeruginosa* with MIC values of 12–150 μM (Knappe et al., 2008). LP2006 derived from *Nocardiopsis alba* has activities against *Enterococcus faecium*, *B. subtilis*, *Bacillus anthracis*, and *Mycobacterium smegmatis*. Anantin B2 exhibits moderate antibacterial activity against *B. subtilis* (MIC of 12.5 μM) (Tietz et al., 2017). Sviceucin presents moderate activity against Gram-positive bacteria, such as *Bacillus megaterium*, *Lactobacillus bulgaricus*, *S. aureus*, *Lactobacillus sakei* (MICs of 1.25–2.5 μM) (Li et al., 2015). Streptomonomicin is active against *B. anthracis*, *Bacillus halodurans*, *B. cereus*, *B. subtilis* (MICs of 4–128 μg/mL or 2–57 μM), which is the causative pathogen of anthrax (Metelev et al., 2015). Ubonodin shows antibacterial activity against several pathogens of the *Burkholderia* genus (Cheung-Lee et al., 2020). Lassomycin derived from *Lentzea kentuckyensis* shows antibacterial activity against a variety of *M. tuberculosis*, *Mycobacterium avium*, and *M. smegmatis* (MICs of 0.1–3.1 μg/mL or 0.07–1.65 μM) (Su et al., 2019). Lariatins produced by *Rhodococcus jostii.* K01-B0171 exhibited selective activity against *M. smegmatis* (MICs of 3.13–6.25 μg/mL; DIZs of 18–19 mm at 10 μg/disk). Additionally, humidimycin identified from *Streptomyces humidus* CA-100629 was characterized as an anti-fungal enhancer of the fungal cell wall inhibitor caspofungin against *Candida albicans* and *Aspergillus fumigatus*, and itraconazole against *A. fumigatus* (Valiante et al., 2015; Sanchez-Hidalgo et al., 2020). It is suggested that the synergistic effect exerted by the drug combination results from the misbalancing of the high osmolarity glycerol signaling pathway and hitting the caspofungin salvage pathway of human-pathogenic fungi.

**TABLE 3:** Bioactivities of known lasso peptides.

Lasso peptide	Native products	Types	Antimicrobial activity against	Protease inhibitory activity	Anti-virus activity	Anti-cancer activity	Peptide antagonist	References
Astexin-1	*A. excentricus*^*a*^	Class II	*C. crescentus –*	–	–	–	–	Maksimov et al., 2012b
Anantin B1	*Streptomyces* sp. NRRL S-146^*b*^	Class II	*E. coli* MIC of 100 μM	–	–	–	Atrial natriuretic factor	Wyss et al., 1991; Tietz et al., 2017
Anantin B2	*Streptomyces* sp. NRRL S-146^*b*^	Class II	*E. coli* MIC of 100 μM *B. subtilis* MIC of 12.5 μM	–	–	–	Atrial natriuretic factor	Wyss et al., 1991; Tietz et al., 2017
BI-32169	*Streptomyces* sp. (DSM 14996)^*b*^	Class III	*–*	–	–	–	Glucagon receptor	Knappe et al., 2010
Capistruin	*B. thailandensis* E264 ^*a*^	Class II	*B. caledonica, B. caribensis, B. ubonensis, B. vietnamiensis, E. coli, P. aeruginosa* MICs of 12–150 μM	RNA polymerase	–	–	–	Knappe et al., 2008
Chaxapeptin	*S. leeuwenhoekii* strain C58^*b*^	Class II	*S. aureus, B. subtilis* MICs of 30–35 μg/mL	–	–	Human lung cancer cell line A549	–	Elsayed et al., 2015
Lariatins	*Rhodococcus* sp. K01-B0171^*b*^	Class II	*M. smegmatis* MICs of 3.13–6.25 μg/mL; DIZs of 18–19 mm at 10 μg/disk	–	–	–	–	Iwatsuki et al., 2007
Lassomycin	*L. kentuckyensis*^*a*^	Class II	*M. tuberculosis, M. avium, M. smegmatis* MICs of 0.1–3.1 μg/mL or 0.07–1.65 μM	–	–	–	–	Su et al., 2019
LP2006	*N. alba*^*b*^	Class IV	*E. faecium, B. subtilis, B. anthracis, M. smegmatis* MIC of 6.25–50 μM	–	–	–	–	Tietz et al., 2017
Microcin J25	*E. coli* AY25^*a*^	Class II	*E. coli, S. newport, S. enteritidis, S. flexneri, E. bugandensis* MICs of 0.01–5 μg/ml	RNA polymerase	–	–	–	Salomon and Farias, 1992; Pati et al., 2018
Propeptin	*Microbispora* sp. SNA-115*^*b*^*	Class II	*P. aeruginosa, M. phlei, X. oryzae* DIZs of 10.6–14.5 mm at 40 μg/disk	Prolyl endopeptidase	–	–	–	Kimura et al., 1997
RES-701	*Streptomyces* sp. RE-701^*b*^	Class II	*–*	–	–	–	Endothelin receptor B	Tanaka et al., 1994
Siamycin-type*	*Streptomyces* sp. strains^*b*^	Class I	Gram-positive bacteria (Supplementary Table 1)	HIV-1 aspartyl protease/myosin light chain kinase	Anti-HIV	–	–	Potterat et al., 1994; Constantine et al., 1995
Streptomonomicin	*S. alba*^*b*^	Class II	*B. anthracis, B. halodurans, B. cereus, B. subtilis, L. monocytogenes, E. faecalis, S. aureus* MICs of 4–128 μg/mLor 2–57 μM	–	–	–	–	Metelev et al., 2015
Sungsanpin	*S. sannurensis*^*b*^	Class II	*–*	–	–	Human lung cancer cell line A549	–	Um et al., 2013
Sviceucin	*S. sviceus* DSM 924T^*b*^	Class I	*E. faecalis, B. megaterium, L. bulgaricus* 340, *S. aureus* subsp. *aureus* ATCC 6538, *L. sakei* subsp. *sakei* DSM20017, *Streptomyces* sp. 523 MICs of 1.25–2.5 μM	–	–	–	–	Li et al., 2015
Ulleungdin	*Streptomyces* sp. KCB13F003^*b*^	Class II	*–*	–	–	Human lung cancer cell line A549	–	Son et al., 2018
Ubonodin	*B. ubonensis* MSMB2207^*a*^	Class II	*B. cepacia* MICs of 4 μM *B. multivorans* MICs of 31 μM	–	–	–	–	Cheung-Lee et al., 2020

*^a^Proteobacteria, ^b^Actinobacteria; ^∗^Siamycin I/II, RP71955, aborycin, MS-271 and humidimycin were summarized as siamycin-type lasso peptide for their high similarity. A. excentricus, Asticcacaulis excentricus; C. crescentus, Caulobacter crescentus; E. coli, Escherichia coli; B. subtilis, Bacillus subtilis; B. thailandensis, Burkholderia thailandensis; B. caledonica, Burkholderia caledonica; B. caribensis, Burkholderia caribensis; B. ubonensis, Burkholderia ubonensis; B. vietnamiensis, Burkholderia vietnamiensis; P. aeruginosa, Pseudomonas aeruginosa; S. leeuwenhoekii, Streptomyces leeuwenhoekii; S. aureus, Staphylococcus aureus; M. smegmatis, Mycobacterium smegmatis; L. kentuckyensis, Lentzea kentuckyensis; M. tuberculosis, Mycobacterium tuberculosis; M. avium, Mycobacterium avium; N. alba, Nocardiopsis alba; E. faecium, Enterococcus faecium; B. anthracis, Bacillus anthracis; S. newport, Salmonella newport; S. enteritidis, Salmonella enteritidis; S. flexneri, Shigella flexneri; E. bugandensis, Enterobacter bugandensis; M. phlei, Mycobacterium phlei; X. oryzae, Xanthomonas oryzae; B. halodurans, Bacillus halodurans; B. cereus, Bacillus cereus; L. monocytogenes, Listeria monocytogenes; E. faecalis, Enterococcus faecalis; B. megaterium, Bacillus megaterium; L. bulgaricus, Lactobacillus bulgaricus; L. sakei, Lactobacillus sakei; B. cepacia, Burkholderia cepacia; B. multivorans, Burkholderia multivorans.*

### Enzyme Inhibitory Activity

Lasso peptides with the protease inhibitory activities are presented in Table 3. The most important enzyme inhibitory activity of lasso peptides, e.g., MccJ25 (Delgado et al., 2001), citrocin (Cheung-Lee et al., 2019b), capistruin (Kuznedelov et al., 2011), klebsidin (Metelev et al., 2017), is the inhibition of the RNA polymerase in Gram-negative bacteria. Darst et al. demonstrated that MccJ25 and capistruin block trigger-loop folding of bacterial RNA polymerase (RNAP) by binding within RNAP secondary channel, thus inhibiting the RNAP function (Braffman et al., 2019). Besides, siamycin-type lasso peptide, MS-271, isolated from *Streptomyces* sp. inhibited the smooth muscle myosin light chain kinase (Yano et al., 1996). Propeptin produced by *Microbispora* is a prolyl endopeptidase (PEP) inhibitor with weak antibacterial activity against *P. aeruginosa*, *Mycobacterium phlei*, and *Xanthomonas oryzae* (Kimura et al., 1997).

### Antiviral Activity

It was observed in previous studies that lasso peptides possess antiviral effects (Table 3). The siamycin-type lasso peptides from *Streptomyces* like siamycin I (MS-271, BMY29304)/II (BMY 29303) (Constantine et al., 1995; Tsunakawa et al., 1995; Yano et al., 1996), aborycin/RP 71955 (Potterat et al., 1994), humidimycin (Valiante et al., 2015; Sanchez-Hidalgo et al., 2020), specialicin (Kaweewan et al., 2018) are most frequently described. They differ from one another only at position 4 (Val or Ile), 8 (Asp, Asn, and Val) or17 (Val or Ile). In position 8, humidimycin is the only one having an aspartic residue, and specialicin is the only one having a valine residue (Supplementary Table 1). The siamycin-type lasso peptides except humidimycin have been found to inhibit HIV infection *in vitro* (De Clercq, 2000). Aborycin/RP71955 is similar to HIV protease inhibitors in terms of its activities. Hence, it suppresses normal assembly of HIV by inhibiting the activity of the HIV protease, thus suppressing HIV (Potterat et al., 1994). The HIV envelope glycoprotein gp41 appears to be the most likely target for the mode of action of siamycin-type lasso peptides (Constantine et al., 1995). They might act by preventing the oligomerization of the HIV transmembrane glycoprotein gp41 or by interfering with interactions between gp41 and the envelope glycoprotein gp120, the cell membrane, or the membrane-bound proteins (Constantine et al., 1995). The exact mechanism of action of siamycin-type lasso peptides remains unsolved.

### Peptide Antagonist and Antitumor Activity

Anantin is a peptide antagonist of the atrial natriuretic factor (Wyss et al., 1991). RES-701 and BI-32169 were reported as antagonists of the endothelin receptor B and the glucagon receptor, respectively (Tanaka et al., 1994; Knappe et al., 2010). Lasso peptides are also active against certain tumor types; for example, chaxapeptin and sunganpin, class II lasso peptides from *Streptomyces leeuwenhoekii* and *Streptomyces sannurensis*, respectively, can inhibit the invasion of human lung cancer cells (Um et al., 2013; Elsayed et al., 2015). The class II lasso peptide ulleungdin exhibited a significant inhibitory effect on cancer cell invasion and migration (Son et al., 2018). One lasso peptide may possess multiple biological activities. Chaxapeptin not only had antimicrobial activity but also exhibited inhibitory activity in a cell invasion assay with human lung cancer cell line A549 (Elsayed et al., 2015).

## Potential Application of Lasso Peptides

### Therapeutical Potential of Lasso Peptides

Much attention has been paid on the lasso peptides for their diverse bioactivities, such as antimicrobial, antitumor, and receptor antagonistic activities, many of which are medically relevant.

The co-evolution and coexistence of bacteria, archaea, eukarya, and viruses in the human gut has led to the development of specialized antimicrobials (Baquero et al., 2019; Garcia-Gutierrez et al., 2019). MccJ25, isolated from baby feces, has displayed robust antimicrobial behavior (Salomon and Farias, 1992). Moreover, MccJ25 can neutralize endotoxins and suppress the production of inflammatory cytokines through immune regulation. The positive effect of MccJ25 in terms of preventing intestinal damage and inflammatory response caused by enterotoxin ETEC K88 (Yu et al., 2018) suggests that it could be applied as a preventative drug to reduce pathogenic infection in animals, food, or humans.

Due to the slow development of anti-tuberculosis (TB) drugs and the widespread occurrence of antibiotic resistant strains of *M. tuberculosis*, TB remains a severe and problematic infectious disease. Lassomycin exhibits remarkable activity against multidrug-resistant *M. tuberculosis*. The bactericidal mechanism of lassomycin stimulates the ATPase activity of ClpC1 but uncouples it from ClpP1P2-dependent proteolysis (Su et al., 2019). Lariatin A/B also shows notable anti-TB activity by targeting *M. tuberculosis* cells (Zhu et al., 2019). The discovery and molecular structure of lassomycin and lariatin A/B can inspire the development of new promising therapies for TB. Humidimycin was characterized as a new synergist of the fungal cell wall biosynthesis inhibitor caspofungin (Valiante et al., 2015). The lack of cytotoxicity of humidimycin alone suggests its possible use as a combined treatment of invasive fungal infections (Valiante et al., 2015; Sanchez-Hidalgo et al., 2020). Based on the assessment of PEP levels in the postmortem brain of patients with Alzheimer’s disease, a strong correlation was established between the increase in PEP activity and Alzheimer’s disease (Aoyagi et al., 1990). Propeptin with PEP-inhibitory activity is a candidate for antiamnestic drug (Park et al., 2006). RES-701, as an endothelin B receptor-selective antagonist, may serve as potential therapeutics against cardiovascular disease, renal disease, and asthma (Katahira et al., 1995). BI-32169 is the antagonist of the glucagon receptor and used for treating diabetes (Knappe et al., 2010). The lasso peptides sungsanpin, chaxapeptin, and ulleungdin that exhibit an inhibitory effect on cancer cell invasion, are used for the development of anti-cancer drugs and the analysis of tumor metastasis (Um et al., 2013; Elsayed et al., 2015).

### Natural Scaffold for Epitope Grafting of Short Peptides or Amino Acids

Altering precursors through mutagenesis of the corresponding gene is a common strategy to engineer lasso peptides and their analogs. Commonly, the sequence variations in the core regions are tolerated by the processing enzymes during modification, which enables diversification of lasso peptides.

**FIGURE 4:**
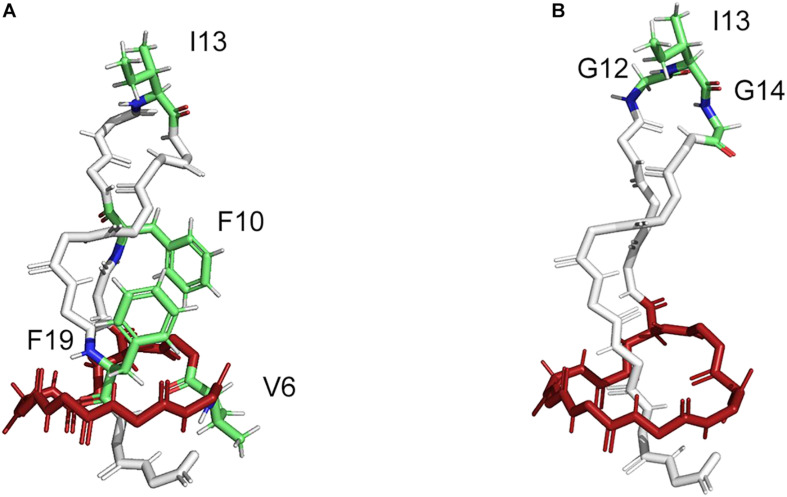
Structure of MccJ25 with indicated modifiable positions (PDB code: 1Q71). **(A)** The modifiable positions (V6, I13, F10, and F19) in the core peptide region of MccJ25 as a natural scaffold used for grafting four non-canonical amino acids (ncAAs) (Piscotta et al., 2015). **(B)** The modifiable positions (G12-I13-G14) in the core peptide region of MccJ25 as natural scaffold used for grafting the bioactive RGD peptide epitope (Knappe et al., 2011).

In this way, several lasso peptides have been successfully modified for therapeutic applications. The structure of MccJ25 comprises three distinct features; the macrolactam ring (residues 1–8), the loop (residues 9–19), and the tail (residues 20–21). Val6 within the macrolactam ring region and the residues Phe10, Ile13, and Phe19 in the loop region of MccJ25 were chosen for the substitution with four non-canonical amino acids (ncAAs) (Figure 4A) (Piscotta et al., 2015); the MccJ25 scaffold showed the remarkable tolerance against the substitutions by ncAAs. Moreover, 16 MccJ25 variants carrying ncAAs retained measurable antimicrobial activity, providing alternatives for the introduction of non-proteinogenic amino acids (Piscotta et al., 2015). Besides, supplementation-based incorporation (SPI) and stop-codon suppression (SCS) approaches were successful for co-translational incorporation of isostructural and orthogonal ncAAs into the lasso peptide capistruin (Al Toma et al., 2015). Three different positions in the core region of the capistruin were chosen for the incorporation of ncAAs. These positions are located in the “ring” (Gly4), in the “loop” (Ala10), and in the “tail” (Gly17) region of the peptide. This further exemplifies that the ribosomal peptides containing ncAAs can be processed from the precursor peptide stage by the post-translational biosynthetic machinery to yield mature peptide derivatives.

The modifiable position G12-I13-G14 in the loop region of MccJ25 did not affect the lasso structure in the biosynthesis (Figure 4B) (Knappe et al., 2011). The bioactive RGD peptide epitope was grafted into the MccJ25 scaffold to produce a nanomolar integrin inhibitor with affinities for α_*v*_β_3_, α_*v*_β_5_, and α_5_β_1_ integrins, which inhibited the capillary formation in cell culture assays. The structural alignment of the native MccJ25 and the grafted MccJ25 illustrated that the RGD substitution did not alter the overall structure of the molecular framework, displaying the robustness of MccJ25 as a scaffold for epitope grafting of short peptide sequences (Knappe et al., 2011). In addition, it is possible to generate lasso peptide-protein fusions by joining the end of a lasso peptide precursor to a protein and co-expressing it with the processing enzymes. In this way, Link and co-workers accomplished the generation of fusions of mature astexin-1 lasso peptide to the N-terminus of GFP and a leucin zipper protein (Zong et al., 2016). Furthermore, the chemically-synthesized macrolactam ring from RES-701-1 was coupled to some bioactive peptides, thereby were combining activities and enhancing stabilities (Shibata et al., 2003). Hybrid peptides of RES-701-1 as endothelin B receptor selective antagonist and endothelin could be applied as the promising therapeutic candidates against pancreatic adenocarcinoma (Shibata et al., 1998; Cook et al., 2015). These indicate that segments of lasso peptides can be biologically active and useful even outside a lasso peptide topology. Thus, recombinant genetics and chemical reaction were employed to design lasso peptides with highly thermal and proteolytic stability for use as drug carriers and molecular probes in medical applications.

## Conclusion and Prospects

Lasso peptides are a class of ribosomally assembled natural products. They possess various biological activities, such as antimicrobial activity, enzyme inhibition, receptor blocking, anticancer properties and HIV antagonism (Potterat et al., 1994; Tanaka et al., 1994; Constantine et al., 1995; Iwatsuki et al., 2007; Li et al., 2015; Valiante et al., 2015). This wide range of interesting activities keeps motivating researchers to identify novel lasso peptides through employing genome mining. Lasso peptides can be obtained by isolation from natural hosts or from heterologous production. The strategies for heterologous production of lasso peptides have been comprehensively summarized in this review, highlighting their benefits as being time saving, environment-friendly, and economical. However, the large-scale production of lasso peptides has not yet been achieved. Also, the libraries of lasso peptide heterologous expression systems including synthesis methods and producing strains need to be significantly expanded. Despite extensive experimental and computational studies of lasso peptides (Hegemann et al., 2015; Tietz et al., 2017; Su et al., 2019), the regulation of the production of lasso peptides and deciphering the roles of lasso peptides in nature are great challenges for the next years.

Based on the diversity of their biological activities, lasso peptides could be excellent examples for the treatment of gastrointestinal diseases, tuberculosis, Alzheimer’s disease, cardiovascular disease, fungal infections and cancer. Lasso peptides are suitable molecular backbones for epitope grafting, owing to their thermal and proteolytic stability (Hegemann, 2019). Modified lasso peptides could be applied as molecular probes or as drug carries for therapeutic applications (Knappe et al., 2011; Piscotta et al., 2015; Zong et al., 2016). However, these modifications also lead to lowered production levels (Zimmermann et al., 2013, 2014; Hegemann et al., 2016; Zong et al., 2017). Therefore, further investigation of lasso peptides should focus on the potential application of these emerging natural peptides as promising candidates for medical use and on uncovering important factors that are affecting the stability and kinetics of the unthreading process of lasso peptides.

## Author Contributions

CC wrote the manuscript. Z-CH revised this manuscript thoroughly. Both authors contributed to the article and approved the submitted version.

## Conflict of Interest

Z-CH collaborated with Jiangsu Target Pharma Labs Inc. on scientific research, such as fermentation; but was not employed by the company. The remaining author declares that the research was conducted in the absence of any commercial or financial relationships that could be construed as a potential conflict of interest.
