# Supplementary material for: Lasso Peptides: Heterologous Production and Potential Medical Application
Source: Front Bioeng Biotechnol. 2020 Sep 28;8:571165. doi: 10.3389/fbioe.2020.571165 (PMC7549694; doi:10.3389/fbioe.2020.571165)
Supplement: Supplementary file 1 [file Data_Sheet_1.docx]

**Table S1: Comparation of the siamycin-type lasso peptides. The differences in the leader peptides and core peptides are highlighted.**

| **Siamycin-type** | **Native products** | **Sequence** | **Antimicrobial activity against** | **Enzyme inhibitory activity** | **Anti-**  **virus activity** | **References** |
| --- | --- | --- | --- | --- | --- | --- |
| Humidimycin  (MDN-0010) | *S. humidus* CA-  100629 | MSAIYEPPALQEIGDFDELTK-CLGIGSCDDFAGCGYAIVCFW | The potentiating effect of caspofungin against  *A. fumigatus and*  *C. albicans*  The potentiating effect of itraconazole against *A. fumigatus* | - | - | (Valiante et al., 2015; Sanchez-Hidalgo et al., 2020) |
| Aborycin  /RP71955 | *Streptomyces* sp. SCSIO ZS0098  *Streptomyces* sp*.* 9440 | MTAIYEPPALQEIGDFDELTK-CLGIGSCNDFAGCGYAVVCFW | *B. subtilis* ATCC 6633  *B. brevis* ATCC 9999  *S. aureus* ETH 2070  *P. saccharophila* ATCC 15946  *S. viridochromogenes* TÜ 57 | HIV-1 aspartyl protease | Anti-HIV | (Potterat et al., 1994) |
| Siamycin I  /MS-271  (BMY29304) | *Streptomyces*  sp. AA6537  *Streptomyces* sp. M-271 | MSAIYEPPMLQEVGDFEELTK-CLGVGSCNDFAGCGYAIVCFW | *B. subtilis*  *E. faecium*  *S. aureus* | Myosin light chain kinase | Anti-HIV | (Tsunakawa et al., 1995; Yano et al., 1996) |
| Siamycin II  (BMY29303) | *Streptomyces* sp. AA6532 | MTAIYEPPALQEIGDFDELTK-CLGIGSCNDFAGCGYAIVCFW | *Enterococci*  *S. aureus* | - | Anti-HIV | (Constantine et al., 1995) |
| Specialicin | *S. specialis* JCM 16611T | MSATTVYEPPALQEIGDFDELTK-CLGVGSCVDFAGCGYAVVCFW | *M. luteus* | - | Anti-HIV | (Kaweewan et al., 2018) |

(**Abbreviations**:

**Continued**

*A. fumigatus: Aspergillus fumigatus; C. albicans: Candida albicans; B. subtilis: Bacillus subtilis;*

*B. brevis: Bacillus brevis; S. aureus: Staphylococcus aureus;*

*P. saccharophila: Pseudomonas saccharophila; S. viridochromogenes: Streptomyces viridochromogenes;*

*E. faecium: Enterococcus faecium; M. luteus:Micrococcus luteus*)
